# The impact of cultural frame switching on wellbeing- systematic review

**DOI:** 10.1371/journal.pone.0332701

**Published:** 2025-09-24

**Authors:** Eshia Garcha, Amber Qureshi, Ciarán O’Driscoll, Madiha Shaikh

**Affiliations:** Research Department of Clinical, Educational and Health Psychology, University College London, London, United Kingdom; Paris School of Business, FRANCE

## Abstract

**Objective:**

This pre-registered review synthesised findings from studies examining individuals’ experiences of cultural frame switching (CFS) and its potential impact on well-being.

**Background:**

CFS is a strategy used by bi/multicultural individuals to navigate daily life by shifting between cultural frameworks in response to environmental cues, influencing thoughts, emotions, and behaviours.

**Method:**

Eleven studies were identified through systematic searches across four electronic databases, with a total sample size of 2,657. The JBI approach to qualitative synthesis was applied, alongside evidence synthesis for the quantitative findings.

**Results:**

The findings suggest that CFS may influence well-being in both positive and negative ways. Potential benefits include enhanced personal relationships, workplace well-being and success, cultural adjustment, and identity navigation. However, CFS may also contribute to identity conflict and associated distress, lower satisfaction with life, perceived discrimination, reduced self-esteem, diminished authenticity, and increased psychological symptoms such as anxiety and low mood.

**Conclusions:**

While CFS appears to have both beneficial and adverse effects on well-being, the limited research in this area makes it difficult to draw definitive conclusions. Further investigation is needed to better understand these mechanisms, why certain individuals experience more challenges, and how to mitigate potential negative impacts. Expanding research in this area could inform future models of support for individuals from minority and multicultural backgrounds.

## Introduction

Cultural frame switching (CFS) is a process that involves activating one culture’s knowledge structures (i.e., cultural frame) in response to contextual cues,thus switching between two cultures in response to the environment and situational and cultural cues [1–3]. It is a dynamic psychological process through which people actively fit aspects of themselves including, self-concept, emotions, and behaviours, to the surrounding cultural context. This can involve adjusting speech/language, appearance, expression, and behaviours using internalised cultural frames which are meaning systems of learned ideas, knowledge, values, beliefs, relevant to a specific culture [4]. These frames can impact thoughts, feelings, and behaviours [5]. The alternation model [6] suggests that individuals can connect with both cultures, without losing their cultural identity, and do this through CFS and do not have to choose or value one over the other, reducing stress and anxiety.

Biculturalism is a broad construct that refers to individuals who have a mixed racial or ethnic identity; have lived in more than one country or immigrated to another country; have parents from different cultures; lived in multicultural societies; have had significant or lengthy exposure to two cultures or self-label/categorise themselves as bi/multicultural [7]. Research suggests that bicultural individuals differ in the way that they respond to the same cultural context; some may reject the culture that they are in, and others may assimilate to the specific culture [8]. Although bicultural individuals may differ in the way they integrate and navigate their two or more cultures, biculturalism is considered an ideal acculturation strategy and leads to improved benefits in wellbeing and adjustment, due to being able to navigate both cultures [9]. However, biculturals may also face the dilemma of trying to navigate and combine two differing or conflicting cultures [6].

The transformative theory of biculturalism [10] suggests that bicultural individuals use three processes to negotiate their cultures, which in turn impacts their experiences and characteristics of being bicultural (in addition to direct influence from their culture). These processes are hybridising, integrating, and frame-switching. Thus, frame switching can have an impact on sense of self, motivation, and cognition as it fosters greater flexibility of the self, allowing shifts in line with the cultural context, whilst maintaining consistency within contexts. This may provide a less demanding way of navigating identities in daily life compared to the complexity of integration. However, frame-switching can also create conflict, leading to a sense of detachment when aspects of oneself are compartmentalised.

Cheng et al. [11] proposed an Integrative Psychological Model of Biculturalism which explains cultural frame switching as one of the psychological processes influenced by the interaction of individual factors, contextual factors such as cultural context and cultural cues, and individual bicultural identity, which in turn impacts psychological, social, and behavioural adjustment. Differences in the interacting factors such as cultural context (being in the mainstream or heritage culture) may influence different bicultural identification and hence influence different use of psychological processes (CFS, motivation, perception of self-concept, cognitive complexity, bicultural knowledge access) and related outcomes. An individual may respond differently in different cultural context due to factors such as, generation, length in second culture, value conflict, engagement with culture and political context.

Frame-switching as a strategy for managing multicultural identity involves two styles: hybridising, where individuals blend desirable elements of different cultures to shape their identity, and alternating, where they switch between cultural identities based on context (CFS). Both styles influence psychological, social, and behavioural adjustment. Ward et al. [12] suggested that alternating identity style predicted greater identity conflict and poor psychological adaption, as perceptions that parts of yourself are contradictory can cause feelings of conflict and confusion. Frame switching can lead to depleted cognitive resources, hindered performance, lower feelings of authenticity [13], lower psychological wellbeing, relationship quality, adjustment, self-esteem and more stress, anxiety, and depression [10] and are perceived as less authentic [14]. Molinsky [15] argues that performing a frame switch can cause value conflict resulting in guilt, distress, and anxiety.

However, research also indicates the CFS can have a positive impact on wellbeing. It can be adaptive [13] and is important for professional development, wellbeing, protection from discrimination, identity consolidation [16], promotes connection with both cultures [6], satisfies need for belonging [17] and leads to increased cognitive complexity [18].

A pre-registered (PROSPERO 2022 CRD42022352791) synthesis of studies that explore individual’s experience of CFS and the impact this has on their social wellbeing was conducted. The construct of social wellbeing used in the study is informed by literature highlighting how CFS can lead to negative outcomes, lower self-esteem and satisfaction with life [19,20], lower self-reported authenticity [21], and higher levels of perceived discrimination, anxiety, and low mood [22]. Understanding the impact of CFS could contribute to supporting the wellbeing of those from a bi/multicultural background.

## Method

### Aim

The aim of this review was to identify and synthesise studies exploring how an individual’s wellbeing is impacted by CFS.

### Design

The Joanna Briggs Institute Approach to qualitative synthesis [23] was used for qualitative findings and the best evidence synthesis approach was used to synthesise quantitative findings.

### Search method

Systematic searches of PsycINFO, MEDLINE, EMBASE, and Web of Science were conducted in October 2024. Given the limited research in this area, the search strategy focused on “cultural frame switching” and “culture switching,” with studies reporting on its impact identified through the inclusion criteria and screening process. To ensure a comprehensive approach, search terms specifically targeting impact were omitted, as well-being and adjustment were broadly defined; instead, the focus remained solely on cultural frame switching.The following combination of search terms was used: cultur* switch* OR cultur* frame switch* OR (cultur* and frame and switch*) OR (cultur* adj2 switch*) OR (cultur* adj2 (frame and switch*)).

There was no limit on the publication date.

Studies were included if the following criteria were met:

- Studies that explored the experience of CFS (which was defined as subconsciously switching between two or more cultures in response to the environment. This involved adjusting speech, appearance, expressions and/or behaviours, using cultural frames which both reside within the individual), and its outcomes/impact on wellbeing and adjustment broadly (psychological, general or social wellbeing).
- Participants of all ages were included.
- Studies published in English.

Studies were excluded if:

- They referred only to the process of (linguistic) code-switching (specific to language adjustments only) as the aim is to explore the broader psychological and cultural aspects of CFS and how these influence well-being. Code-switching, which refers specifically to language adjustments in bilingual or multilingual contexts, often emphasises language use rather than the deeper shifts in cultural frames of reference that are integral to CFS.
- Studies that did not contain results specifically on cultural frame switching and its impact on wellbeing; studies that reported only the outcomes of biculturals outside the context of cultural frame-switching.
- Other systematic reviews; theoretical papers; books and presentations.

### Study selection process

**Fig 1:**
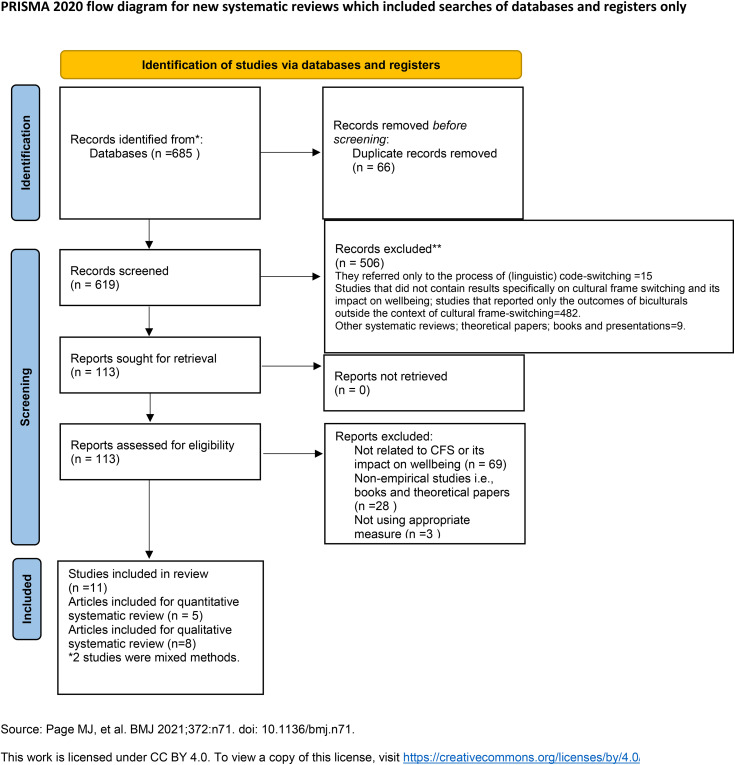
PRISMA 2020 flow diagram for literature search.

The review was undertaken and reported in compliance with the Preferred Reporting Items for Systematic Reviews and Meta-Analyses (PRISMA) 2020 guidelines [24].The process is summarised in Fig 1. The total number of records retrieved by combining the searches was 685. Eleven studies met the final inclusion criteria (six qualitative, three quantitative studies and two mixed methods). This resulted in a total of eight qualitative findings and five quantitative findings.

Studies identified in the search were exported to a reference management software and articles, were independently screened by EG and AQ based on the inclusion and exclusion criteria. Firstly, title and abstracts were screened for eligibility and were excluded if they did not meet inclusion criteria. Secondly, all remaining full texts were independently screened for eligibility by the same two reviewers. No disagreements were observed.

### Quality appraisal tool

The Joanna Briggs Institute (JBI) Critical Appraisal Checklist for Qualitative Research [25] and the JBI Checklist for Analytical Cross-Sectional Studies [26] were used to assess risk of bias.

### Data extraction process

JBI Manual for Evidence Synthesis tool for extracting data was used. Data extracted included author, journal, year of publication, data on the purpose/aim of research, methodology, method, phenomena of interest (CFS), setting, geographical, culture/ethnicity, participants (sample size and demographics), data analysis, conclusion, findings (in relation to CFS and wellbeing), and illustrations (for qualitative studies).

### Data synthesis process

Meta-synthesis of qualitative findings used the JBI meta-aggregative approach. Based on Cochrane Group guidance on qualitative meta-syntheses [27] this approach was appropriate as the aim was to summarise and aggregate data in relation to the specific review question on understanding the impact of CFS on wellbeing, rather than re-interpreting primary findings. It also allowed accommodation of studies from varied qualitative and philosophical methods. In this approach, relevant study results are translated to ‘findings. ‘Findings’ are extracts of the author’s interpretation of their results. ‘Findings’ were identified through repeated readings of text in the results section of studies. The ‘findings’ were accompanied by ‘illustrations’ – direct quotes of the participant’s voice, observation, or other supporting data. The ‘findings’ were aggregated to create ‘categories’ based on similarity in meaning. ‘Categories’ are defined as brief descriptions of a key concept arising from the aggregation of two or more similar findings. These ‘categories’ were then combined into synthesised findings to answer the review question.

A best evidence synthesis was used to critically consider quantitative findings. This focused only on CFS (or alternating) groups in all studies as opposed to other identity profiles. Due to the heterogeneity of group samples, method and measures, and a lack of responses from authors, a meta-analysis was not deemed appropriate.

### Researcher background and preconceptions

The primary researchers, who have a bicultural background (British and Punjabi) and experience CFS, used bracketing as an ongoing reflective method to mitigate the impact of preconceptions. This involved setting aside assumptions and expectations about the topic [28], re-reading papers and results to ensure key findings were not overlooked, and critically reflecting on their own beliefs.

### Description of study characteristics

The characteristics of the 11 included studies are presented in Table 1 for qualitative findings and Table 2 for quantitative findings and summarised below.

**Table 1:** Study characteristics for qualitative studies.

Study	Aims	Sample	Country	Setting	Cultures	Data collection method	Data analysis method
Barros and Albert (2020)	To further understand how individuals deal with living within two different cultural frames and thus identities in their daily lives and interactions from their own individual narratives.	10 Portuguese migrant parent-adult child pairs. 10 parents (7 females, 3 males) and 10 Adult Children (9 females, 1 male).	Luxembourg	Portuguese families in Luxembourg	Portuguese and Luxembourgish	Semi-structured interview	Narrative approach and thematic analysis
Bohon (2016)	To explore if and how the model of self-authorship aligns with Chinese undergraduates’ students’ psychological development and in what ways do traditional Chinese cultural constructs influence Chinese undergraduates meaning making and journey toward self-authorship	12 Chinese Undergraduates from China studying in the US (6 female, 6 male)	USA	Mid-Western US university	Chinese and American (US)	Semi-structured interview and focus groups	Grounded Theory
Carmichael et al. (2022)	To explore the experience of genetic counselling students who identify with a racial or ethnic minority group	32 genetic counselling students who identify as an ethnic minority (28 females, 4 males)	USA	Online in the US	American (US) and various self-identified cultures (Asian, Indian, Hispanic/Latinx, White, Black, or Black African American, Other)	Focus groups	Constructivist Grounded Theory
Qumseya (2018)	To qualitatively investigate participants lived experiences of their cultural identity styles within their socio-political and family contexts.	18 young people (10 in New Zealand, 8 in Israel; 10 females, 8 males)	New Zealand and Israel	Online (Israel) or in-person (New Zealand)	New Zealand – NZ and Arab Israel- Israeli and Palestinian	Interview	Thematic Analysis
Richardson (2021)	To investigate expatriate adjustment experiences of biculturals (individuals with two internalized cultural profiles) in a multicultural host country	14 bicultural expatriate executives (6 females, 8 males)	Malaysia	Senior management workplaces in Kuala Lumpur and Penang	Malaysian and Various cultures	Semi-structured interview	Blended approach to identify both shared and unique themes and experiences that emerged from the interviews to complement the a priori themes
Rincon and Hollis (2018)	To explore how are cultural code-switching strategies woven into Chicana/o college students’ experiences while persisting through a majority four-year college environment?	12 Chicano/a students	USA	Colorado higher education institutions	Chicano/a and American (US)	Semi-structured interview	Hermeneutic phenomenological process
Steel and Heritage (2020)	To understand the experiences of Indigenous employees in mainstream Australian workplaces.	10 Indigenous Australian employed in mainstream Australian workplaces. (5 females, 5 males)	Australia	Metropolitan mainstream Australian workplaces	Indigenous Australian – Australian	Semi-structured interview	Thematic Analysis
Stuart and Ward (2011)	To explore acculturation experiences of Muslim youth in New Zealand	216 Muslims in New Zealand- check this	New Zealand	Leadership development workshops	New Zealand- Muslim	Self-report measures, interview, and focus groups	Thematic Analysis

**Table 2:** Study characteristics of quantitative studies.

Study	Aims	CFS sample	Country	Cultures	CFS measure	Wellbeing measure
Barros & Albert (2020)	To examine the methods in which second-generation participants of Portuguese origin juggle an environment with both of their cultures	21	Luxumbourg	Portuguese and Luxembourgish	Bicultural identity orientation scale-revised (Comănaru, 2009)	Self-esteem (Rosenberg, 1965). 10 items on a 5-point Likert scale (1 = *totally disagree* to 5 = *totally agree*) e.g., *“I think I have a number of good qualities.”* SWL (Diener et al., 1985). 5 items rated on a 7-point Likert scale (1 = *do not agree*; 7 = *fully agree)* e.g., *“I am satisfied with my life.”*
Firat & Noels (2022)	To explore the cultural interactions and experiences of participants from immigrant backgrounds	1143	Canada	Canadian and one of the following: Chinese, Indian, Filipino, Pakistani, Korean, Vietnamese, Nigerian, Lebanese, Ukrainian, British, German or Polish	Bicultural identity orientation scale (Comănaru et al., 2018)	Perceived discrimination (Taylor et al., 1990). 7 items on a 6-point Likert scale (1 = *never*, 6 = *always*) *Participants rated the extent to which their ethnic group experienced discrimination by Canadians due to racial characteristics for example.* Depression and Anxiety Stress Scale (Lovibond & Lovibond, 1995). 21 items on a 4-point Likert scale (1 = *never*, 4 = *almost always*) e.g., *“I felt that life was meaningless.”*
Jack (2018)	To explore two mechanisms used in negotiating cultural identities: hybridising and alternating and how they are related to mental health	870	USA	American and Hispanic	Multicultural Identity Styles Scale (Ward et al., 2018)	Centre for Epidemiologic Studies Depression Scale (Radloff, 1977). 1 item on a 6-point Likert scale (1 = *strongly disagree* to 6 = *strongly agree*) e.g., *“I have felt down and unhappy today.”*
West et al. (2018)	To explore whether frame switching makes participants feel less authentic and therefore decreasing their wellbeing	43	USA and Canada	American or Canadian, and either White, Mixed, Black, East Asian, Latin American, South Asian, Native or other	Participants were instructed to describe a situation where they were with one cultural group but their behaviour would have been different if were with their other cultural group	State authenticity (Lenton et al., 2013). 12 items on 7-point Likert scale (1 = *strongly disagree* to 7 = *strongly agree*) e.g., *“I behaved in accordance with my values and beliefs.”*
Qumseya (2018)	To explore hybrid and alternating identity styles and whether these styles have different effects on wellbeing	143	New Zealand and Israel	New Zealand and Arab	Multicultural Identity Styles Scale (Ward et al., 2018)	Perceived discrimination (Noh & Kaspar, 2003). 7 items measured on a 5-point Likert scale (1 = *never* to 5 = *very often*) e.g., *“I have been treated disrespectfully.”* SWL (Diener al., 1985). 5 items on a 5-point Likert scale (1 = *strongly disagree* to 5 = *strongly agree)* e.g., “*So far I have got the important things I want in life.”* Psychological symptoms (Berry et al., 2006). 15 items on a 5-point Likert scale (1 = *never* to 5 = *very often*) e.g., *“I worry a lot of the time.”*

*SWL: satisfaction with life.

### Participants

#### Findings from qualitative studies.

The sample size ranged from 10 to 216, with 334 participants in total. Although not all studies reported gender, most reported participants were female. Participants were based in the US [29–33], Luxembourg [19], New Zealand [12,34], Israel [12], Malaysia [35], and Australia [36]and identified across various cultures. Age of participants was not consistently reported.

#### Findings from quantitative studies.

The sample size ranged from 70 to 1143, with 2323 participants in total. Participants were based in Luxembourg [19], Canada [22,37], America [29–31,37]and New Zealand [12] however ethnicity and cultural background varied greatly including people who identified as Portuguese, Chinese, Indian, Filipino, Pakistani, Korean, Vietnamese, Nigerian, Lebanese, Ukrainian, British, German and Polish, Hispanic, Mixed, Black, East Asian, Latin American, South Asian, and Arab.

### Data collection and analysis methods

#### Qualitative studies.

Five of the eight studies used only interviews to collect data; one study used only focus groups; one study used interviews and focus groups and one study used interviews, focus groups and open space self-report surveys. The studies used various methods of analysis, although the most common was thematic analysis (four of the eight studies). All analysis methods involved the generation of themes.

#### Quantitative studies.

Common self-report measures were used to capture CFS. These included the Bicultural Identity Orientation Scale [38], the Bicultural Identity Orientation Scale-Revised [39] and the Multicultural Identity Styles Scale (MISS) [12]. One study primed participants to think about a situation where they had engaged in CFS and no scale was used to measure CFS [40].

## Results

### Quality appraisal of qualitative studies

The results of the quality appraisal are presented in the appendix/supplementary material.

#### Congruity of research methodology.

There was clear congruence between the stated philosophical stance and the chosen methodology in studies that explicitly reported their theoretical framework and adopted a methodological approach consistent with this stance (e.g., hermeneutic phenomenology combined with in-depth qualitative interviews exploring individual perspectives). Similarly, there was alignment between the research methodology and research questions, where the chosen approach was appropriate for addressing the research aim (e.g., using semi-structured interviews with bicultural expatriates to examine their adjustment process). Furthermore, all qualitative studies demonstrated consistency between methodology and data collection methods. However, congruence between methodology and representation of analysis was evident in only five of the eight qualitative studies. In three studies studies [16,36,41], this aspect was rated as unclear due to a lack of detailed explanations regarding the analysis, despite the stated type of analysis. Where methodological details or representation of analysis were insufficiently described, the assessment was marked as ‘unclear.’

#### Researcher influence.

Four of the eight studies explicitly located the researcher(s) within a cultural and theoretical context [16,29,31,35] and discussed the potential influence of the researcher on the study. One study [29] acknowledged the positive rapport established with participants but did not elaborate on the potential influence of the researcher on data collection or analysis.

#### Participants voice.

Seven studies effectively represented participants’ voices by including illustrative quotes to support findings and conclusions. However, in one study [35], this aspect was rated as unclear, as participant quotes were not presented for each theme discussed.

#### Ethics.

Four studies explicitly reported obtaining ethical approval from a research ethics committee [20,36,41,42]. One study [31] discussed ethical considerations and the use of informed consent but did not explicitly state whether formal ethical approval had been obtained. The remaining three studies [16,29,35] made no reference to ethical approval.

### Quality appraisal of quantitative studies

Four studies clearly specified inclusion and exclusion criteria for their sample. One study [32] did not explicitly state inclusion criteria but described participant demographics in detail. Based on the sample description, it can be inferred that the inclusion criteria likely included Hispanic and American participants. All studies provided detailed descriptions of the study sample, setting, demographic characteristics, and cultural identity.

Objective, standardised measures were used to assess both CFS and well-being in four studies. One study [40] did not include a direct measure of CFS but instead primed participants to recall an instance in which they engaged in CFS. All studies identified potential confounding factors and employed appropriate statistical analyses.

### Meta-synthesis of qualitative findings

**Fig 2:**
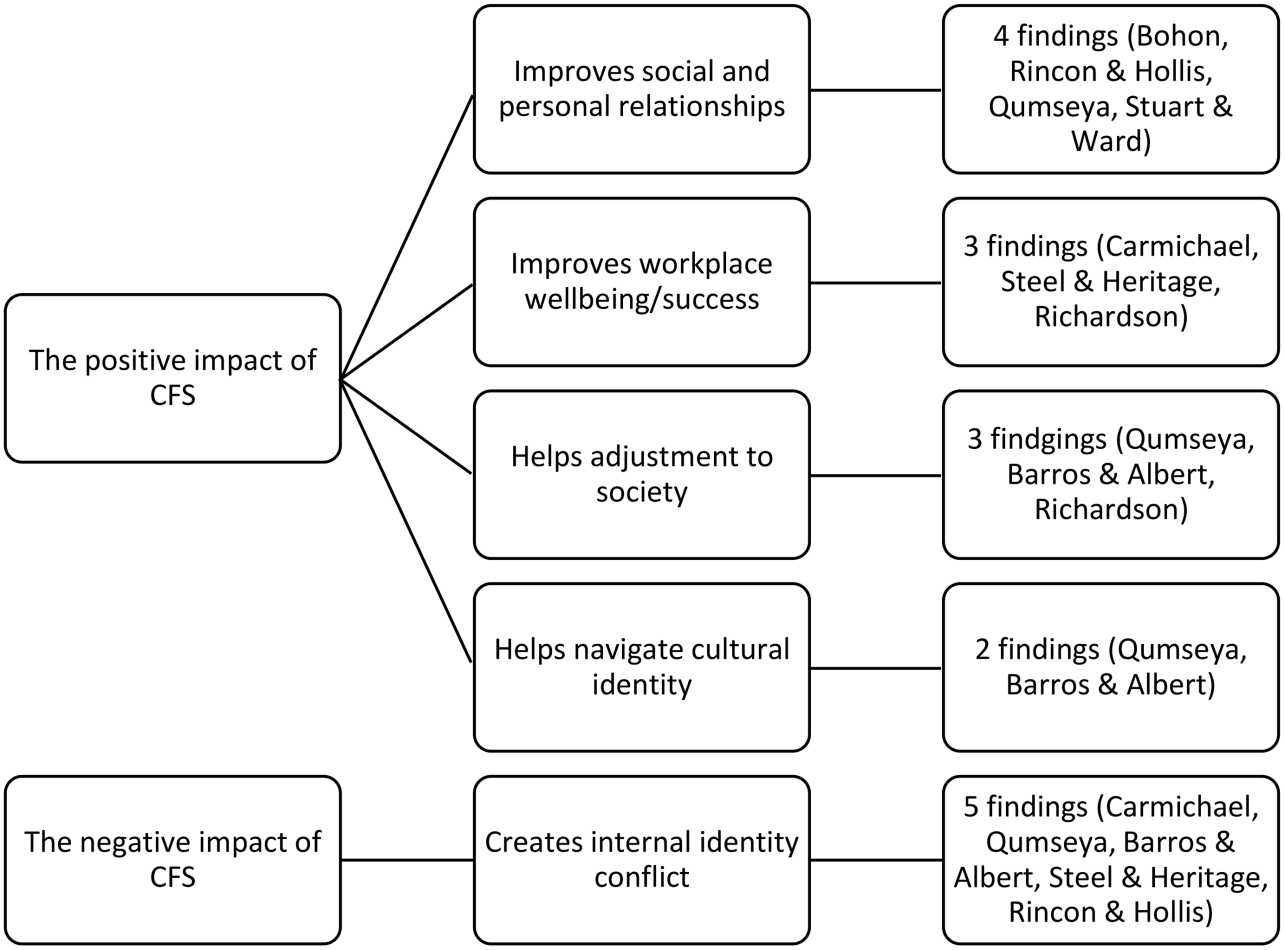
Synthesised findings and categories for qualitative studies.

Analysis of the eight studies identified 20 findings, which were organised into five categories and two synthesised findings: the positive and negative impacts of Cultural Frame Switching (CFS) on well-being (see Fig 2). All eight studies highlighted positive impacts of CFS, which were synthesised into four categories: improving social and personal relationships, enhancing workplace well-being and success, aiding adjustment to society, and helping individuals navigate cultural identity. In contrast, five of the eight studies reported a negative impact, synthesised into one main category: creating internal identity conflict. This finding suggests that CFS can generate tension between different identities, leading to distress and a sense of not belonging, as if a part of oneself is lost. Additionally, two studies identified a fear of adopting harmful habits and the expression of emotion as part of the negative impact of CFS. These findings were independent of others and therefore could not be categorised or synthesised as such and are discussed separately.

### The positive impact of CFS

#### Category 1: Improves social and personal relationships.

Improved social and personal relationships was a recurring theme in four studies. CFS maximised social benefit as choosing between two selves allowed navigation between cross cultural relationships [31].

*“The hard point is, how can you balance your Chinese background and the things you learn here? …It’s kind of a paradox. It’s like, how should you behave and what’s the best way for you to do things here? …It’s like, how to … convey our Chinese background but other times, not to keep too far away from those United States students and try to fit into with them?”*

This study also described how switching between two cultures had improved efficiency, relations and communication in teams and university.

CFS was “a useful communication tool” and allowed participants to fit in and not “stand out” in university settings [29].

*“When you’re able to have those conversations, it’s a lot different. I mean it is really difficult to have those conversations in a classroom with all White people. So, I think code-switching happened with the types of conversations I was having and the way that I was having them. But the words were always the same. It was more like directing conversations.”*

*“I paid attention to my body language and everything so that I can make sure that not only do I appear in my mind to people that I belonged here but exuded that in my actions and body language.”*

CFS not only allowed maintenance of social relationships with friends but also “deepened extended family relationships” [20].

*“I feel like people do that as a way to fit in so obviously at home you would be fitting in with your family but outside you would be fitting in with your friends and your colleagues…”*

This study suggests that switching between cultures gives people freedom to identify with various cultural identities by context, whilst managing cultural expectations, therefore being able to make and maintain social and familial connections.

CFS aids balancing competing expectations, social norms and navigating tension based on the environment. Importantly, avoiding conflict [16].

*“It’s just a matter of putting the spotlight on which aspect (is important) depending on the context, it’s a matter of placing relevance in the context. I play different roles, everybody does.”*

#### Category 2: Improves workplace wellbeing and success.

The second category described the positive impact on wellbeing in the workplace, this was discussed in three of the eight studies. CFS allows for improvement in rapport and confidence in the workplace, which also created a sense of empowerment for the individuals [42].

*“My supervisor really appreciated how I used that bonding moment with her to do some genetic counselling- related things, and she told me that I should use that as my strength moving on, which was a really good thing to hear towards the beginning of my rotation. Made me feel confident.”*

Whilst Steel and Heritage [36] found that the agility of switching between cultures was a strength as it facilitates navigation of intercultural tension in the workplace.

*“The language changes, the body language changes, the way we sit...back in the time and even now when Aboriginal people acted and spoke and live like White people, they got a big tick of approval so when we put on that (White) hat that means that we step in out of who we are into this other frame like an acting frame, that’s what we’re doing, we’re acting.”*

CFS led to better adjustment at work, with no notable difficulties reported [35].

*“You’ve got to understand that people work differently in other countries [outside the US] and so you’ve really got to be patient and try not to be too [pausing to think] “American” [...] Growing up in an Indian family and partly in India itself, I understand this and so when I arrived here, I was very careful not to come over as this American guy who wants to change how everything works here [...] You’ve got to adjust – in my case, it wasn’t that difficult because I just switched on my Indian button, as it were!”*

#### Category 3: Helps adjustment to society.

Another category that emerged from three studies was helping adjustment to wider society.

Richardson [35] identified a theme of ‘Adjustment outside work’.

*“No major difficulties were reported when it came to non-work adjustment. As with work-related adjustment, this was attributed in large part to the participants’ ability to switch cultural frames”.*

In New Zealand alternating between cultures helped participants to adjust to the majority societal culture whilst maintaining family relationships at home and without negative consequences [20].

*“…you have to adapt to your certain environment in order to…adjust. So, when I come home, I try to just make things in order to have fun with my parents…. and have a good time. You have to obviously do what they enjoy as well…for example to actively speak in Arabic…when I come home, I completely let go of my New Zealand culture or identity and I try to stick…primarily to my…Arabic identity…just it’s easy, easier to relate to my family and it makes them more comfortable as well.”*

CFS also aided adjustment to receiving countries, and a stronger connection was reported with the receiving culture, however, connection was maintained with the home culture [41].

*“I feel more LU than PT, but I don’t leave out my roots. […] we went once a year to Portugal, we stayed a month like almost all PTs […] At home, we spoke PT, it’s not that I don’t feel PT, but I feel more LU because of living my whole life here...”*

Qumseya [20] found that alternating between cultures helped one of the participants achieve their goals. In this example, the participant wished to advocate on their university campus, but needed to connect with the majority culture to do so and wanted to do this whilst retaining their internal identity.

*“when I want to demand for collective rights…I would connect with their [majority culture] organisations in different ways too, I would speak differently, and therefore I work on spreading the rights for all and spreading awareness about our identity.”*

Therefore, switching allowed her to connect with the majority audience, whilst achieving her goals of spreading awareness and demanding rights for her minority identity.

#### Category 4: Helps navigate cultural identity.

CFS allowed individuals to identify with both or all cultural frames they held at different times rather than feeling as if they had to lose one or be in conflict with each other, making it easier and less distressing to navigate society.

CFS meant that individuals did not identify with just one culture, but felt connected to both of their cultures [41]..

*“I feel both things, it’s a mixture of both things […] Pfff I don’t know what I am after all […] I’m not connected to the PT culture, but I’m not connected to the LU culture either, it’s both […] It depends on the situation […] it depends where I am with whom I am…”*

CFS allows for maintenance of identity when connecting with people from both cultures [20].

*“I feel de jure in this day, society people are more judgmental. So, me splitting my Arab mentality and my Westernized mentality creates so much easy approach to make friends and [makes it] easy to get along…I’m not being two people, someone who I’m not.”*

### The negative impact of CFS

#### Category 1: Creates internal identity conflict.

CFS could cause conflict between behaviour expected in workplace cultures, compared to behaviour expected by culturally concordant patients [42]. One participant found a clash between her two cultural frames when speaking with a patient from the same background as herself.

*“They’re in their seventies, they’re older. And you’re supposed to be really respectful and deferential. You don’t talk about things like cancer and breasts… There isn’t even a word for ovary in Punjabi. It was tough to balance that, being Indian and being respectful and not talking about things like that versus doing my job.”*

Other studies also identified a conflict between cultures, which felt like a loss or repression of identity and self [20,29,36]. Here, participants reported feeling expected to repress part of their identity and so it felt devalued, resulting in distress and loss of part of themselves.

*“You would lose your sense of self. Many times, I would wonder about who I am, who should I go to?…they are different people.…Maybe I do not want to be one of them, not everything in me is compatible with them….It is very difficult” [* 20 *]*

*“From one side, it…makes me live in a struggle with myself in terms of not showing who I really am. This is a negative.” [* 20 *]*

*“To work with the White man, you got to act like the White man, and you assimilate, and then you distance yourself from your identity, and your people, and your culture...this is how mainstream has made it, for them to fail their culture to rise to the top levels within the Non-Aboriginal culture, that they’ve got to leave their own behind and put on that White cap.” [* 36 *]*

*[cultural code-switching is] “unauthentic.” [* 29 *]*

A cultural identity conflict can arise from feeling a lack of belonging caused by CFS [41]. This was expressed by several second-generation adult children in their study.

*“I look like an alien there, right? Because here [Luxembourg] I’m PT and there [Portugal] I’m LU. I still don’t have a country.”*

### Additional findings

#### Fear of adopting harmful habits.

The first was CFS leading to a fear of adopting harmful habits [20]. Participants who identified with an alternating identity style identified disadvantages of alternating including fear of assimilating and losing key cultural values by engaging in detrimental behaviour from the second or dominant culture, such as the use of alcohol.

*“Negatives are when one takes bad things from these experiences. But that is not me; I take good things. I will not make mistakes; I will not drink [alcohol].”*

#### Expression of emotion.

The second uncategorised finding related to cultural switching repressing the ability to express emotions freely (Rincon and Hollis, 2018), where a participant felt that switching to the dominant university culture meant a barrier to her emotional expression.

“I’d have to tone it back and couldn’t be so explicit with my emotions... with my feelings. That anything you...any racial issue that you have is just because you’re too sensitive or you’re being... you want to be coddled.”

### Evidence synthesis for quantitative studies

The quantitative evidence consistently indicated a negative impact of CFS on well-being. The findings were synthesised across six overlapping well-being domains: satisfaction with life (SWL), perceived discrimination, self-esteem, state authenticity, and psychological symptoms (including low mood, anxiety, and psychosomatic symptoms). Notably, Firat and Noels [22], Jack [32], and Qumseya [20]used the term ‘low mood’ instead of ‘depression’ to avoid implying a clinical diagnosis.

Effect sizes and 95% confidence intervals (CIs) were calculated for all studies except West et al.[40], which employed a between-group analysis due to its study design. The remaining studies used a within-group analysis. Where effect sizes were not reported, r was calculated using an online effect size calculator (Wilson, n.d.), based on the sample size and means (M) and standard deviations (SD) of the CFS and well-being measures. CIs were subsequently derived using a separate online calculator. (see table 3).

**Table 3:** Summary of descriptive analysis for five quantitative studies.

Study	Wellbeing measure	N	Effect size and 95% CIs
**Barros & Albert (2020)**	Self-esteem SWL	21	*r * = −.95 95% C.I. = [0.8700, 0.9783] *r * = −.97 95% C.I. = [0.9151, 0.9861
**Firat & Noels (2022)**	Perceived discrimination Low mood Anxiety	1143	*r * = .20 95% C.I. = [0.1437, 0.2550] *r * = .17 95% C.I. = [0.1131, 0.2258] *r* = .16 95% C.I. = [0.1030, 0.2160]
**Jack (2018)**	Low mood	870	*r * = .37 95% C.I. = [0.3113, 0.4261]
**Qumseya (2018)**	Perceived discrimination SWL Psychological symptoms (low mood, anxiety & psychosomatic symptoms)	143	*r * = .30 95% C.I. = [0.1429, 0.4424] *r* = −.11 95% C.I. = [−0.2693, 0.0551] *r * = .23 95% C.I. = [0.0684, 0.3798]

*SWL: satisfaction with life.

### Satisfaction with life

With regard to SWL, two papers used Diener’s scale [43] to measure this. Barros and Albert [41] demonstrate an association between CFS and SWL. Furthermore, Qumseya [20] found that the alternating identity style was significantly related to lower SWL.

### Perceived discrimination

Two papers measured and discussed perceived discrimination in relation to CFS. Firat and Noels [22] used a scale by Taylor et al. (1990) and found that perceived discrimination was associated with CFS. Moreover, Qumseya [20] used a scale by Noh and Kaspar (2003) and found a positive correlation between perceived discrimination and CFS.

### Self-esteem

Although there was limited data exploring self-esteem, it was measured in one study using Rosenberg’s [44] scale. Barros and Albert found that that CFS participants reported intermediate levels of self-esteem.

### State authenticity

State authenticity is defined as the subjective sense of being one’s true self [45] and was measured in one paper using Lenton et al.’s [45] scale. West et al. [40] found that those in the CFS condition reported significantly less authenticity (**M* *= 4.37, *SD *= 1.17) than control (**M* *= 5.29, *SD *= 0.94), and no switching conditions (**M* *= 4.99, *SD *= 1.23), and that the control and no switching conditions did not significantly differ on authenticity.

### Psychological symptoms

Psychological symptoms were measured in three studies.

Firat and Noels [22] found that CFS was positively correlated with low mood and anxiety. Jack [32] found a significant positive relationship between increased CFS and low mood, and Qumseya [20] found that depression, anxiety and psychosomatic symptoms appeared to deteriorate with CFS.

## Discussion

This review explores the positive and negative impacts of Cultural Frame Switching (CFS) on the well-being of bicultural and multicultural individuals. Notably, positive effects were reported exclusively in qualitative studies and encompassed four key areas: improving social and personal relationships, enhancing workplace well-being and success, facilitating societal adjustment, and aiding in navigating cultural identity. In contrast, negative impacts were identified across both qualitative and quantitative studies. These were characterised by lower life satisfaction, higher perceived discrimination, reduced self-esteem, diminished authenticity, increased psychological symptoms (including low mood, anxiety, and psychosomatic complaints), and lower overall psychological well-being. Additionally, CFS was found to contribute to an internal identity conflict.

The divergence between qualitative and quantitative findings may be attributed to methodological differences. Qualitative research is particularly suited to capturing the nuanced and context-dependent nature of individuals’ experiences, whereas quantitative studies often rely on standardised measures that may not fully encapsulate the complexities of cultural adjustment. In this review, qualitative findings predominantly emphasised social adaptation and contextual flexibility, whereas quantitative findings focused on conventional well-being indicators such as life satisfaction, psychological well-being, and distress, which are commonly used to assess overall adjustment. This distinction highlights the importance of integrating multiple research approaches to gain a more comprehensive understanding of how CFS influences well-being.

### Positive impact of CFS

The findings suggest that CFS serves as an adaptive strategy, facilitating navigation between cultural contexts and interpersonal relationships across various settings (e.g., workplace, university, friends, and family). This aligns with the transformative theory of biculturalism [10,46], which posits that bicultural individuals employ frame switching to align with contextual norms while maintaining consistency within specific cultural environments. Additionally, Cheng et al. [11] support this through their integrative psychological model of biculturalism, which highlights the role of contextual and environmental cues in guiding CFS. However, while these theoretical frameworks underscore the adaptive nature of CFS, they do not fully address the potential cognitive and emotional costs associated with frequent frame switching, which warrants further investigation [8].

The thematic categories ‘Helps adjustment to society’ and ‘Helps navigate cultural identity’ align with the alternation model [6], which posits that successfully alternating between cultural frames allows individuals to integrate both cultural identities without diminishing either. This process has been linked to increased authenticity, self-esteem, and psychological well-being [7]. However, it is noteworthy that only three of the eight studies contributed to the category of adjustment, and only two studies supported cultural identity navigation. While the diversity of the participant samples (e.g., Portugal/Luxembourg, New Zealand/Arabic, Israeli/Palestinian, Malaysian/Various) suggests that these benefits may be generalisable, the limited number of studies necessitates caution in drawing broad conclusions. Future research should incorporate longitudinal designs to assess whether the positive effects of CFS persist over time.

The category ‘Improves social and personal relationships’ was the most frequently supported finding, with four studies reporting improvements across diverse cultural groups (e.g., Chinese, American, Chicano/a, New Zealand, Arabic, Israeli, Palestinian). LaFromboise et al. [6] argue that individuals who engage in CFS experience reduced stress and anxiety, which may facilitate relationship building. Participants commonly described shifting frames between peer and familial environments, reinforcing the argument that CFS is context-dependent [15]. However, while these findings highlight the potential benefits of CFS, they do not address whether prolonged engagement in frame switching leads to emotional exhaustion or cognitive strain, an issue raised in recent acculturation research [47].

The review also found evidence for the positive role of CFS in workplace wellbeing and success. The transformative theory [48] suggests that frame-switching enhances self-flexibility, allowing individuals to maintain consistent identities within specific cultural contexts, including professional environments. This adaptability may foster authenticity and professional success [13]. Given the significant role of work in overall well-being, the ability to navigate cultural contexts effectively may contribute to job satisfaction, professional growth, and social integration [49]. However, while workplace-based studies highlight the advantages of CFS, they often fail to account for power dynamics, microaggressions, and discrimination that bicultural individuals may encounter in professional settings [50], which could complicate the purported benefits.

### Negative impact of CFS

Despite its adaptive potential, CFS was also associated with significant identity conflict, with five of the eight studies contributing to this category. This finding is consistent across diverse cultural samples, suggesting that identity conflict is a common challenge among individuals engaging in CFS. Participants described difficulties in balancing cultural expectations, understanding their identity, and experiencing feelings of inauthenticity. Molinsky’s [15] model of psychological toll suggests that identity conflict arises when personal values and cultural norms are misaligned during frame-switching, leading to distress, anxiety, and guilt. Additionally, the transformative theory of biculturalism [48] and the theory of multicultural identity styles [51] suggest that compartmentalisation of cultural identities can lead to psychological detachment and internal contradictions, exacerbating identity conflict and reducing psychological well-being. However, these models largely overlook the intersectionality of cultural identity with other social identities (e.g., ethnicity, race, religion), which may compound the negative effects of CFS [22].

The review also identified a link between CFS and reduced self-esteem, perceived discrimination, and diminished authenticity. The acculturation complexity model [52], proposes that bicultural individuals experience higher levels of cultural dissonance, which may contribute to internal conflict. Studies by Jack [32] and West et al. [40]found that individuals in CFS conditions reported lower authenticity, reinforcing the psychological burden associated with frequent frame switching. Furthermore, Firat & Noels [22]and Qumseya [20]reported a small effect between CFS and perceived discrimination, whereas Barros & Albert [19]found a large effect on self-esteem, suggesting heterogeneity in the impact of CFS on psychological well-being. These findings indicate the need for further research to disentangle the mechanisms by which CFS affects self-perception and mental health outcomes.

Additionally, the review found that CFS was associated with higher levels of psychological symptoms, contradicting the alternation model [6], which suggests that frame-switching reduces stress. Instead, multiple studies reported increased symptoms of depression and anxiety in individuals engaging in CFS [20,32]. While some research suggests a weak association between CFS and psychological distress [22], others highlight a stronger connection, necessitating further investigation into potential mediating factors, such as social support, resilience, and perceived cultural distance.

### Strengths and limitations

This pre-registered review is the first to systematically assess the impact of CFS on well-being, addressing a critical gap in the literature. The limited number of studies in this area may reflect broader publication bias in research on ethnicity, cultural heritage, and well-being, highlighting the need for further investigation. Despite these constraints, presenting these findings is crucial to drawing attention to this underexplored issue. he use of PRISMA guidelines and independent reviewers strengthens the methodological rigour of the study. The inclusion of the Joanna Briggs Institute tools for quality appraisal enhances the reliability and validity of the findings. Confounding factors are likely to have impacted the findings. For instance, an individual’s socioeconomic status, generation, age, education level or religion may affect well-being as well as CFS experience. Additionally, generational differences may shape the experience of CFS, with younger individuals potentially perceiving frame-switching as a more natural and positive adaptation compared to older generations [53]. Studies which contributed to ‘The positive impact of CFS’ categories were specifically conducted in the workplace [35,36,42], with university students [29,31] or young people [16,20].

Moreover, the duration and intensity of CFS engagement, as well as the cultural distance between the two identities, may moderate its impact on well-being. Future research should explore how sociopolitical contexts and multicultural environments influence the psychological effects of CFS [35].It is known that within different cultures, people may perceive mental health differently [54]. It would be useful for studies to attempt to account for these factors and explore whether there were differences in CFS and outcomes based on whether the cultural context was an individual’s heritage or second culture.

The heterogeneity of study designs and measures used to assess CFS and well-being also complicates comparisons across studies. The reliance on qualitative synthesis (JBI meta-aggregation) means that only findings supported by direct participant quotes were included, potentially underrepresenting broader themes present in the primary studies. Furthermore, half of the qualitative studies did not explicitly state their cultural or theoretical frameworks, raising concerns about implicit biases in data interpretation. Addressing these methodological inconsistencies will be crucial for future research seeking to provide a more nuanced understanding of CFS and its implications for well-being. Nonetheless all studies were included to provide an accurate representation of the available knowledge on this topic.

The differences in CFS and wellbeing measures made it challenging to compare results. The heterogeneous nature of studies did not allow for a meta-synthesis. Additionally, the various aspects of cultural frame switching may have different effects on wellbeing outcomes. Our criterion included a broad definition of CFS inclusive of adaptations to thoughts and behaviours. Furthermore, the MISS only refers to two identity styles, hybridising and alternating, not accounting for any others such as blended, separated, complementary, all of which have been well documented. The MISS has also only been validated in a limited set of ethno-cultural samples.

Given the contrast of findings in qualitative and quantitative research regarding impact of CFS on well-being, mixed methods studies and longitudinal methods may be most helpful in capturing a true picture of the CFS construct. Lastly, the search was restricted to the English language. However, no appropriate papers in a non-English language were found during the soft search prior to the systematic search and therefore this is unlikely to have been a considerable limitation.

### Implications

The implications of this review suggest that CFS may have a complex, dual impact on well-being, although the small number of studies makes these findings somewhat preliminary. While the positive impacts of CFS such as improvements in social relationships, workplace success, and identity navigation are evident, these results should be interpreted with caution. If these benefits hold true across broader contexts, there may be a need for greater cultural understanding and acceptance in workplaces, educational institutions, and society at large. Such measures could potentially help individuals feel less conflicted and reduce the psychological strain associated with CFS. Institutions might consider fostering environments that embrace cultural diversity, as this could help mitigate some of the identity conflict and support more authentic self-presentation, though further research is needed to confirm these potential benefits.

The findings regarding perceived discrimination and lowered self-esteem suggest that these challenges might warrant further attention, particularly in developing policies to support bicultural and multicultural individuals. This could include initiatives such as creating networks for minority groups, offering diversity training for stakeholders, and providing support systems for individuals navigating identity-related stress. However, the small number of studies means that these recommendations are based on limited evidence and require further exploration.

Additionally, it may be important for practitioners and researchers to remain mindful that identity conflict arising from CFS could have negative consequences for mental health, potentially contributing to issues like anxiety, depression, and lower self-esteem. While this review suggests such outcomes, more robust research is needed to understand the full extent of these effects. In the meantime, interventions that aim to support individuals in managing the challenges of cultural negotiation may be helpful, though their effectiveness should be evaluated through further research [42].

### Conclusion

This synthesis provides valuable insights into the impact of CFS on well-being, revealing both positive and negative consequences. The positive outcomes—such as improved social relationships, workplace success, and identity adjustment—support existing theories of biculturalism and frame-switching. However, the negative impact of CFS, including identity conflict, lower self-esteem, and reduced authenticity, points to significant challenges for individuals engaging in frame-switching.

The limited research in this area suggests a need for further investigation into the complexities of CFS and its effects on well-being. Understanding these dynamics, particularly the mediating role of individual and contextual factors, is essential for developing interventions that can help mitigate the negative impacts of CFS and promote the well-being of individuals from multicultural backgrounds. Future research should aim to address these gaps, using mixed methods and longitudinal designs, to offer a more nuanced understanding of how cultural negotiation shapes psychological health in diverse societies.

## Supporting information

S1 File Search terms. (DOCX)

S2 File Quality appraisal. (DOCX)

S3 File PRISMA 2020 checklist. (DOCX)

S4 File Screening- reasons for exclusion. (XLSX)
