# Supplementary material for: The impact of cultural frame switching on wellbeing- systematic review
Source: PLoS One. 2025 Sep 24;20(9):e0332701. doi: 10.1371/journal.pone.0332701 (PMC12459798; doi:10.1371/journal.pone.0332701)
Supplement: S1 File — (DOCX) [file pone.0332701.s001.docx]

**S1 Search Terms**

**Ovid Database Search Terms:**

Database: Embase <1980 to 2022 Week 27>, Ovid MEDLINE(R) ALL <1946 to July 14, 2022>, APA PsycInfo <1806 to July Week 2 2022>

Search Strategy:

--------------------------------------------------------------------------------

1     cultur* switch*.mp. [mp=ti, ab, hw, tn, ot, dm, mf, dv, kf, fx, dq, nm, ox, px, rx, ui, sy, tc, id, tm] (77)

2     cultur* frame switch*.mp. [mp=ti, ab, hw, tn, ot, dm, mf, dv, kf, fx, dq, nm, ox, px, rx, ui, sy, tc, id, tm] (47)

3     (cultur* and frame and switch*).mp. [mp=ti, ab, hw, tn, ot, dm, mf, dv, kf, fx, dq, nm, ox, px, rx, ui, sy, tc, id, tm] (306)

4     1 or 2 or 3 (383)

5     (cultur* adj2 switch*).mp. [mp=ti, ab, hw, tn, ot, dm, mf, dv, kf, fx, dq, nm, ox, px, rx, ui, sy, tc, id, tm] (593)

6     (cultur* adj2 (frame and switch*)).mp. [mp=ti, ab, hw, tn, ot, dm, mf, dv, kf, fx, dq, nm, ox, px, rx, ui, sy, tc, id, tm] (103)

7     1 or 2 or 3 or 5 or 6 (847)

8     7 not cell.mp. [mp=ti, ab, hw, tn, ot, dm, mf, dv, kf, fx, dq, nm, ox, px, rx, ui, sy, tc, id, tm] (325)

***************************

**Web of Science Search Terms**

10 (#9) NOT ALL=(CELL*) 360

9 #4 OR #8 728

8 #5 OR #6 OR #7 342

7 ALL=(“cultur* switch*”) 34

6 ALL=(cultur* and frame and switch*) 309

5 ALL=(“cultur* frame switch*”) 24

4 #1 OR #2 OR #3 465

3 TS=(cultur* near/2 switch*) 458

2 TS=(cultur* NEAR/2 frame-switch) 26

1 TS=(cultur* NEAR/2 frame and switch*) 41
