# Supplementary material for: The impact of cultural frame switching on wellbeing- systematic review
Source: PLoS One. 2025 Sep 24;20(9):e0332701. doi: 10.1371/journal.pone.0332701 (PMC12459798; doi:10.1371/journal.pone.0332701)
Supplement: S2 File — (DOCX) [file pone.0332701.s002.docx]

**S2 Quality Appraisal**

**JBI Critical Appraisal Checklist for Qualitative Research**

Table 3. Critical Appraisal Checklist for qualitative studies.

|  | Barros and Albert (2020) | Bohon (2016) | Carmichael et al. (2022) | Qumseya (2018) | Richardson (2020) | Rincon and Hollis (2018) | Steel and Heritage (2020) | Stuart and Ward (2011) |
| --- | --- | --- | --- | --- | --- | --- | --- | --- |
| 1. There is congruity between the stated philosophical perspective and the research methodology | Yes | Yes | Yes | Yes | Yes | Yes | Yes | Yes |
| 2. There is congruity between the research methodology and the research question or objectives. | Yes | Yes | Yes | Yes | Yes | Yes | Yes | Yes |
| 3. There is congruity between the research methodology and the methods used to collect data. | Yes | Yes | Yes | Yes | Yes | Yes | Yes | Yes |
| 4. There is congruity between the research methodology and the representation and analysis of data. | Unclear | Yes | Yes | Yes | Yes | Yes | Unclear | Unclear |
| 5. There is congruity between the research methodology and the interpretation of results | Yes | Yes | Yes | Yes | Yes | Yes | Yes | Unclear |
| 6. There is a statement locating the researcher culturally or theoretically. | No | Yes | Unclear | No | Yes | Yes | No | Yes |
| 7. The influence of the researcher on the research, and vice-versa, is addressed | No | Yes | No | No | Yes | Unclear | No | Yes |
| 8. Participants and their voices are adequately represented. | Yes | Yes | Yes | Yes | Unclear | Yes | Yes | Yes |
| 9. The research is ethical according to current criteria or, for recent studies, there is evidence of ethical approval by an appropriate body | Yes | Unclear | Yes | Yes | Unclear | Unclear | Yes | Unclear |
| 10. Conclusions drawn in the research report appear to flow from the analysis or interpretation of the data | Yes | Yes | Yes | Yes | Yes | Yes | Yes | Yes |

*Table 4.* *JBI Checklist for Analytical Cross Sectional Studies*

| **JBI Checklist** | **Barros & Albert (2020)** | **Firat & Noels (2022)** | **Jack (2018)** | **West et al. (2018)**  *Study 1* | **Qumseya (2018)**  *Study 1* |
| --- | --- | --- | --- | --- | --- |
| Were the criteria for inclusion in the sample clearly defined? | Yes | Yes | No | Yes | Yes |
| Were the study subjects and the setting described in detail? | Yes | Yes | Yes | Yes | Yes |
| Was the exposure measured in a valid and reliable way? | N/A | N/A | N/A | N/A | N/A |
| Were objective, standard criteria used for measurement of the condition? | Yes | Yes | Yes | Yes | Yes |
| Were confounding factors identified? | Yes | Yes | Yes | Yes | Yes |
| Were strategies to deal with confounding factors stated? | No | Yes | Yes | Yes | Yes |
| Were the outcomes measured in a valid and reliable way? | Yes | Yes | Yes | Yes | Yes |
| Was appropriate statistical analysis used? | Yes | Yes | Yes | Yes | Yes |
